# Near-Complete Genome Sequence of a SARS-CoV-2 VOC 202012/01 Strain in Peru

**DOI:** 10.1128/MRA.00069-21

**Published:** 2021-03-25

**Authors:** C. Padilla-Rojas, L. Barcena-Flores, K. Vega-Chozo, M. Galarza-Perez, H. Bailon-Calderon, P. Lope-Pari, J. Balbuena-Torres, M. Huaringa-Nuñez, O. Caceres-Rey, N. Rojas-Serrano

**Affiliations:** a Laboratorio de Referencia Nacional de Biotecnología y Biología Molecular, Instituto Nacional de Salud, Lima, Peru; b Laboratorio de Referencia Nacional de Virus Respiratorio, Instituto Nacional de Salud, Lima, Peru; Queens College CUNY

## Abstract

A near-complete genome sequence was obtained for a novel severe acute respiratory syndrome coronavirus 2 (SARS-CoV-2) variant of concern (VOC) 202012/01 strain obtained from an oropharyngeal swab sample from a Peruvian patient with coronavirus syndrome who had contact with an individual who had recently returned from England.

## ANNOUNCEMENT

The severe acute respiratory syndrome (SARS) that currently affects the world is caused by severe acute respiratory syndrome coronavirus 2 (SARS-CoV-2), which belongs to the *Betacoronavirus* genus of the family *Coronaviridae* (1, 2). An article has recently been prepublished showing that the new variant designated variant of concern (VOC) 202012/01 has a greater transmission capacity, but it has not been shown to be more pathogenic (3).

During surveillance, we detected a female patient with coronavirus syndrome who claimed to have been in contact with a relative who had recently returned from England. This patient was confirmed as being infected with SARS-CoV-2 by quantitative reverse transcription (RT)-PCR using the Xpert Xpress SARS-CoV-2 test on the GeneXpert system (threshold cycle [*C_T_*] values for the E gene and N2 were 17.9 and 19.6, respectively). Viral RNA was purified from 200 μl of viral transport medium using the Maxwell 16 viral total nucleic acid purification kit in a Maxwell 16 instrument (Promega, Madison, WI, USA) following the manufacturer’s instructions. The purified RNA samples were processed by RT-PCR to amplify overlapping fragments, with primers designed using the SARS-CoV-2 genome from a Wuhan isolate, as reported previously (4). This study was approved by the institutional ethics committee at the Peruvian National Institute of Health (project code OI-045-20).

The amplification process used the SuperScript IV One-Step RT-PCR high-fidelity system kit (Invitrogen), and the amplified products were purified with the PureLink PCR purification kit (Invitrogen). The amplified fragments (2 ng) were processed using the Nextera XT DNA library preparation kit, index adapters, and the MiSeq sequencer (Illumina) following the procedure recommended by the manufacturer. The fastq files generated were processed in the Galaxy platform (5) and cleaned using Groomer v1.1.5 and Trimmomatic v0.38.0 (https://usegalaxy.org). Then, the genomes were assembled using SPAdes v3.12.0 and compared to the reference genome using CONTIGuator v2.7.4 (http://contiguator.sourceforge.net). Nucleotide and amino acid variations were detected using Geneious Prime software v2021.0.3. To perform the phylogenetic analysis, other relevant genomes were downloaded from the GISAID database, the genomes were aligned with MAFFT v7 (https://mafft.cbrc.jp/alignment/server), and a phylogenetic tree was built using the maximum likelihood algorithm (based on the GTR model and the gamma distribution) with the program MEGA X v7.0.26 (https://www.megasoftware.net). Default parameters were used for all software unless otherwise specified.

**FIG 1:**
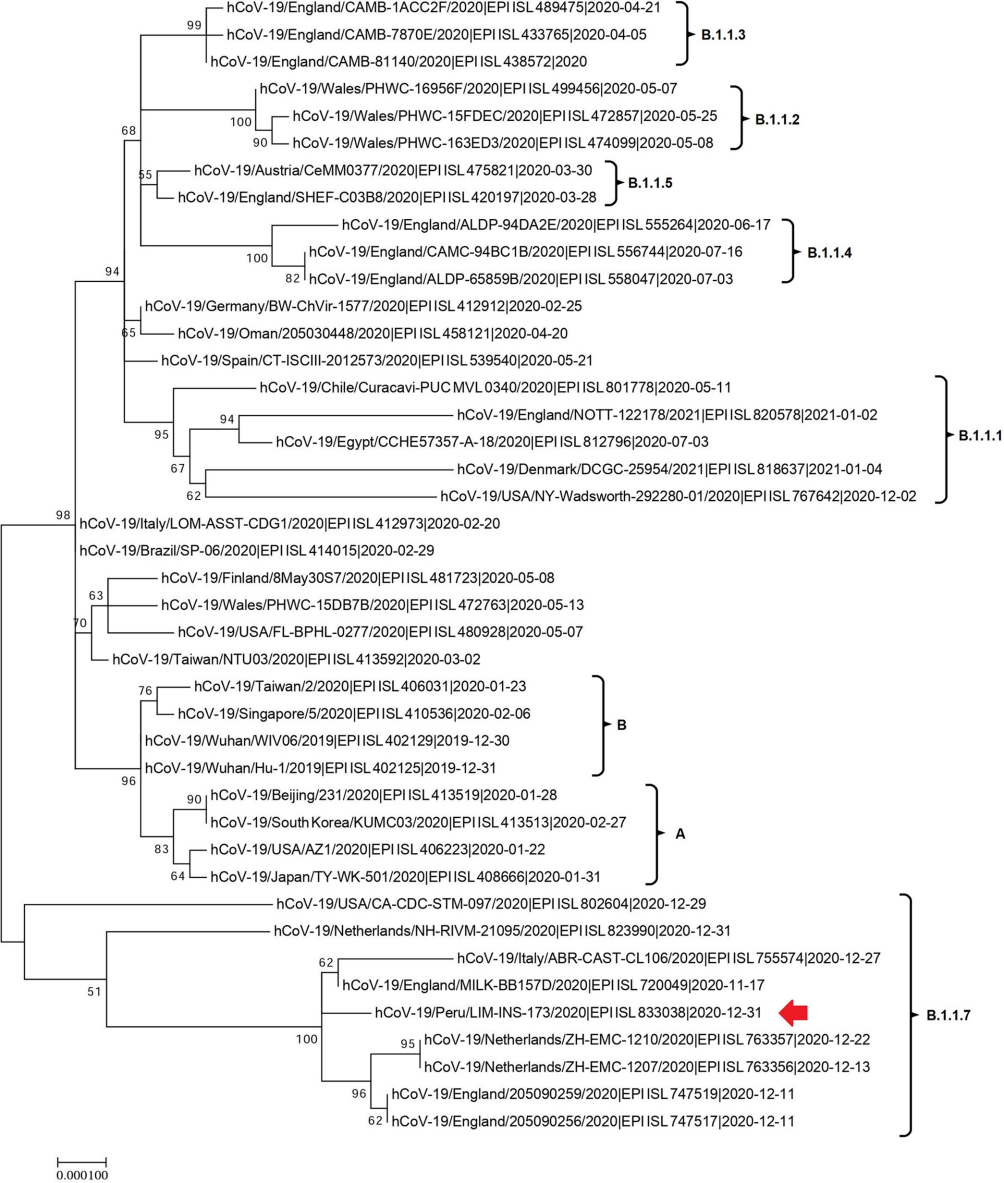
Phylogenetic tree of the VOC 202012/01 strain detected in Peru (indicated by the red arrow) with relevant genomes (the GISAID accession number is indicated in each name). This tree was built with the maximum likelihood algorithm (GTR+G model) using MEGA X.

The genome of this viral isolate has two deletions (aminoacidic position 69 to 70 and position 144 to 145 deletions) and eight mutations (V483I, N501Y, A570D, D614G, P681H, T716I, S982A, and D1118H) in the S gene, similar to VOC 202012/01. Also, it presents the C241T mutation in the 5′ untranslated region, and it presents five nonsynonymous mutations (L730F, T1001I, A1708D, I2230T, and P4715L) and a deletion of three amino acids (S3575, G3676, and F3677) in the *orf1ab* gene. The C27972T mutation, which generates a premature stop, is present in open reading frame 8 (ORF8), while the N gene presents the D3L, D203K, G204R, and S235F variations. The lineage of this sample corresponds to B.1.1.7 according to the Pangolin system, and phylogenetic analysis indicates that it is homologous to the VOC 202012/01 isolates reported in the GISAID database (Fig. 1). This is the first report of this variant in Peru, and a study of the contacts of this patient is being carried out to evaluate how this new variant is spreading.

### Data availability.

The sequence of the SARS-CoV-2 variant was deposited in the NCBI database (GenBank accession number MW494424.1) and the GISAID database (https://www.gisaid.org) (accession number EPI_ISL_833038). The raw reads were deposited in the NCBI Sequence Read Archive (SRA) database (accession number PRJNA623683).
